# Tuberculose de l’omoplate chez l’enfant: à propos d’une localisation exceptionnelle

**DOI:** 10.11604/pamj.2017.28.166.13506

**Published:** 2017-10-20

**Authors:** Driss Hanine, Achraf El Bakkaly, Mohammed Amine Essaoudi, Aiat Allah Skiredj, Abderahmane Malihy, Abdelouahed El Amrani, Mohammed Anouar Dendane, Sidi Zouhair Fellouss El Alami, Tarik El Madhi

**Affiliations:** 1 Service de Chirurgie Orthopédique Pédiatrique, Hôpital d’Enfants de Rabat, CHU Ibn Sina, Faculté de Médecine Mohammed V, Rabat, Maroc; 2 Laboratoire d’Anatomopathologie, Hôpital d’Enfants de Rabat, CHU Ibn Sina, Faculté de Médecine Mohammed V, Rabat, Maroc

**Keywords:** Omoplate, tuberculose, enfant, Scapula, tuberculosis, child

## Abstract

La tuberculose osseuse est une maladie infectieuse qui constitue un véritable problème de santé publique dans les pays en voie de développement où elle sévit de manière endémique. L'atteinte ostéo-articulaire représente 1 à 5% des cas de tuberculose toutes localisations confondues, l'atteinte de l'épaule reste très rare et représente 1 à 2% des localisations ostéo-articulaires. Nous présentons ici un nouveau cas exceptionnel d'une infection tuberculeuse de l'omoplate, atteignant le corps de l'omoplate. La tuberculose est encore un diagnostic différentiel important des maladies rares ou chroniques des os, notamment tumorales. Le diagnostic a été redressé par l'étude anatomopathologique.

## Abstract (en)

Tuberculosis of bone is an infectious disease which represents a major public health problem in developing countries where it is endemic. Osteoarticular involvement accounts for 1-5% of all cases of tuberculosis, shoulder involvement is very rare and accounts for 1-2% of all cases of osteoarticular tuberculosis. We here report an uncommon case of TB infection involving the scapula, reaching the scapular body. Tuberculosis is still an important differential diagnosis of rare or chronic bone diseases, including tumors. In our study anatomopathological examination led to the diagnosis.

## Introduction

La tuberculose ostéoarticulaire représente 1 à 3 % des tuberculoses extra-pulmonaires [1]. La tuberculose de l'épaule constitue la localisation ostéoarticulaire la plus rarement rencontrée. L'atteinte de l'omoplate est extrêmement rare. Son diagnostic est souvent difficile du fait de sa rareté et des pathologies qui peuvent la simuler [2]. Dans notre présent travail, nous rapportons le cas d'une ostéomyélite tuberculeuse de l'omoplate.

## Patient et observation

**Figure 1:**
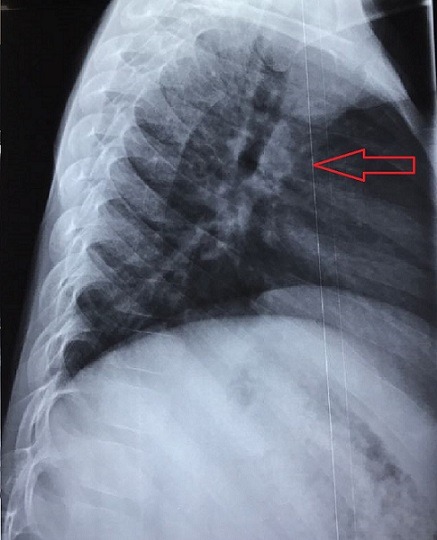
Radiographie standard de l'épaule gauche de profil à l'admission montrant des lésions lytiques au niveau de l'omoplate gauche

**Figure 2:**
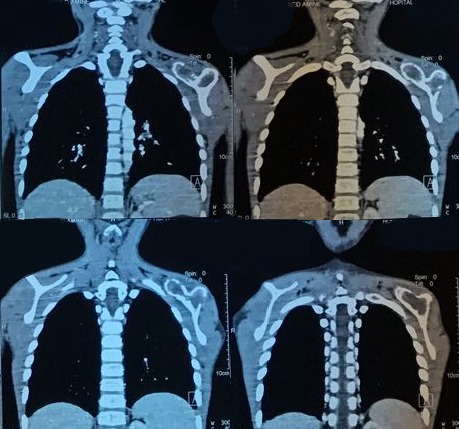
Image scannographique thoracique montrant un processus lésionnel lytique de l'omoplate gauche évoquant en premier un sarcome d'Ewing

**Figure 3:**
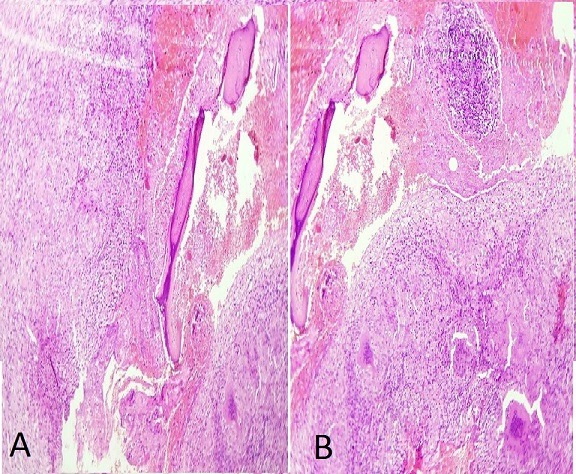
Image histologique de la biopsie osseuse du Scapula montrant l'existence des lésions de granulome à cellules géantes avec des ébauches de nécrose caséeuse (A: grossissement x 10; B: grossissement x 20)

**Figure 4:**
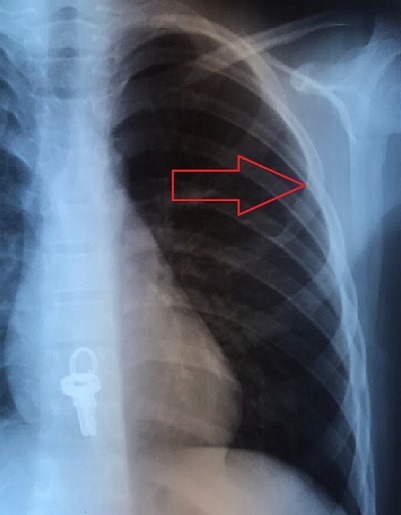
Image de radiographie standard de l'épaule après 4 mois de traitement: on note une reminéralisation de l'omoplate gauche

M.L., âgé de 7 ans a présenté depuis un an suite à la survenue d'un torticolis, une tuméfaction intéressant l'omoplate gauche et augmentant progressivement de volume. Il signalait une fièvre vespérale concomitante et une asthénie. Il présentait aussiun amaigrissement non chiffré. L'examen clinique retrouvait une tuméfaction dure et douloureuse en regard de l'omoplate gauche avec limitation de la mobilité de l'épaule gauche ainsi qu'une douleur à la palpation des apophyses épineuses du rachis dorsolombaire et attitude scoliotique avec gibbosité. Les radiographies standard du rachis ont objectivé une rectitude du rachis et respect du mur postérieur, de la hauteur des corps vertébraux et des espaces inter-somatiques sans lésions ostéolytiques ou ostéocondensantes du rachis. Cependant on note des lésions lytiques au niveau de l'omoplate gauche (Figure 1). La numération sanguine (NFS) montrait un taux de globules blancs normal et une anémie hypochrome, microcytaire à 9.6 g/dl. La vitesse de sédimentation était accélérée à 69 mm à la première heure.La CRP est très élevée à 117.3. Devant l'aggravation de l'état général, une lésion tumorale a été évoquée notamment un sarcome d'Ewing; nous avons complété par une échographie des parties molles de l'épaule gauche qui a montré un processus tumoral lytique de l'omoplate gauche envahissant les parties molles. Puis une tomodensitométrie de l'épaule gauche a été réalisée, objectivant un processus lésionnel lytique de l'omoplate gauche responsable d'une lyse de la corticale avec discrète extension vers les parties molles en regard évoquant en premier un sarcome d'Ewing (Figure 2). Cela motiva une biopsie osseuse dont l'examen histologique mettait en évidence un tissu osseux siège d'un processus granulomateux fait de cellules épithélioides et de cellules géantes de type Langhans ainsi la présence de nécrose caséeuse entourée par des polynucléaires neutrophiles (Figure 3). Vu la fréquence de cette localisation sur terrain d'immunodépression, la sérologie HIV a été demandé pour notre patient et est revenue négative. L'enfant a été mis sous chimiothérapie antituberculeuse avec rifampicine, isoniazide, pyrazinamide et éthambutol pendant deux mois, relayée pendant dix mois par éthambutol et isoniazide. L'amélioration a été spectaculaire. La tuméfaction de l'omoplate a disparu. La vitesse de sédimentation était passée à 20 mm à la première heure. La radiographie de l'épaule montrait des modifications de l'omoplate dans le sens de la guérison avec des images de reminéralisation de l'omoplate (Figure 4). L'examen clinique montrait une restauration de la mobilité de l'articulation de l'épaule et la disparition de l'attitude scoliotique.

## Discussion

L'atteinte de l'épaule représente 1 à 10,5% des formes osseuses de tuberculose, elle est rare [1]. L'atteinte articulaire à Mycobacterium tuberculosis peut se faire soit par voie directe hématogène avec un envahissement de la membrane synoviale, soit par voie indirecte par extension d'un foyer osseux adjacent [1,2]. L'atteinte osseuse primitive peut être difficile à diagnostiquer dans les phases précoces, augmentant le délai diagnostique [3]. Le diagnostic de tuberculose devrait être confirmé par l'isolement de Mycobacterium tuberculosis soit lors de l'analyse histologique, soit par les cultures bactériologiques ou idéalement par les deux [1-3]. Nous avons revu à la fin du 20^ème^ siècle deux grandes séries d'atteinte tuberculeuse ostéoarticulaire extra-vertébrale dans la littérature [4,5]. Les deux possédaient un cas de tuberculose de l'omoplate dans leurs séries, les deuxétaient des lésions uniques. La plupart des autres cas affectent les os longs. L'atteinte tuberculeuse ostéoarticulaire est due au Mycobacterium tuberculosis, et ceci sans prise en considération de l'état immunitaire de l'hôte.L'organisme isolé dans notre cas étant Mycobacteriumtuberculosis, celui-ci étant sensible aux médicaments anti-bacillaires ordinaires, et ceci malgré la présentation extraordinaire et l'âge jeune du patient, comme décrit dans la littérature dans l'étude de Kam [6]. A notre connaissance, notre observation est le premier cas d'une atteinte tuberculeuse de l'omoplate chez un enfant. Le diagnostic est toujours histologique d'où l'intérêt d'une biopsie chirurgicale au moindre doute pour guider l'attitude thérapeutique [2,7]. Après le développement des nouvelles recherches et l'apparition de nouveaux agents chimiothérapiques, l'incidence de cette entité est devenue sous contrôle dans la plupart des pays, mais jamais éradiquée et toujours endémique dans les pays les moins développés [7]. Malheureusement chez nous au Maroc, on reste toujours un pays d'endémie avec surtout les localisations pulmonaires [3]. De nos jours, on retrouve surtout des problèmes de résistance au traitement au monde entier, surtout vu l'augmentation rapide des cas HIV positifs associés [1].

## Conclusion

L'atteinte tuberculeuse des os plats, en dehors d'une atteinte vertébrale, est très rare [2]. Une atteinte de l'omoplate est jusqu'à ce jour non connue et exceptionnelle. Cette infection peut entrainer des dégâts anatomiques importants, sources de séquelles fonctionnelles invalidantes. C'est dire l'importance d'un diagnostic précoce grâce aux prélèvements chirurgicaux pour élucider ces cas cliniques inhabituels. Afin d'éviter tout retard diagnostic, chirurgiens pédiatres et radiologues doivent savoir que la tuberculose peut revêtir les tableaux cliniques et radiologiques de nombreuses pathologies [1,8].

## Conflits d’intérêts

Les auteurs ne déclarent aucun conflit d'intérêt.

## References

[1] Kapukaya A, Subasi M, Bukte Y, Gur A, Tuzuner T, Kilnc N (2006). Tuberculose de l'épaule. Revue du Rhumatisme..

[2] Pertuiset E (2006). Tuberculose ostéoarticulaire extravertébrale. Rev Rhum..

[3] Teklali Y, El Alami ZF, El Madhi T, Gourinda H, Miri A (2003). La tuberculose ostéo-articulaire chez l'enfant (mal de Pott exclu): à propos de 106 cas. Rev Rhum Mal Ostéoartic..

[4] Martini M, Adjrad A, Boudjemaa A (1986). Tuberculous osteomyelitis: a review of 125 cases. Int Orthop..

[5] Vohra R, Kang HS, Dogra S, Saggar RR, Sharma R (1997). Tuberculous osteomyelitis. J Bone Joint Surg Br..

[6] Kam WL, Leung YF, Chung OM, Wai YL (2000). Tuberculous osteomyelitis of the scapula. International Orthopaedics (SICOT)..

[7] Ravinglione ML, Snider DE, Kochi A (1995). Global epidemiology of tuberculosis: morbidity and mortality of a world wideepidemic. JAMA..

[8] Monach PA, Daily JP, Rodriguez-Herrera G, Solomon DH (2003). Tuberculous osteomyelitis presenting as shoulder pain. J Rheumatol..

